# Compact Numerical Aperture 0.5 Fiber Optic Spectrometer Design Using Active Image Plane Tilt

**DOI:** 10.3390/s24123883

**Published:** 2024-06-15

**Authors:** Pinliang Yue, Mingyu Yang, Qingbin Jiao, Liang Xu, Xiaoxu Wang, Mingle Zhang, Xin Tan

**Affiliations:** 1 Changchun Institute of Optics, Fine Mechanics and Physics, Chinese Academy of Sciences, Changchun 130033, China; 2 University of Chinese Academy of Sciences, Beijing 100049, China

**Keywords:** high NA, fiber spectrometer, image plane tilt, achromatism

## Abstract

The numerical aperture of the spectrometer is crucial for weak signal detection. The transmission lens-based configuration has more optimization variations, and the grating can work approximately in the Littrow condition; thus, it is easier to acquire high numerical aperture (NA). However, designing a large aperture focusing lens remains challenging, and thus, ultra-high NA spectrometers are still difficult to acquire. In this paper, we propose a method of setting image plane tilt ahead directly when designing the large aperture focusing lens to simplify the high NA spectrometer design. By analyzing the accurate demands of the focusing lens, it can be concluded that a focusing lens with image plane tilt has much weaker demand for achromatism, and other monochromatic aberration can also be reduced, which is helpful to increase the NA. An NA0.5 fiber optic spectrometer design is given to demonstrate the proposed method. The design results show that the NA can achieve 0.5 using four lenses of two materials, and the MTF is higher than 0.5 when the spectral dispersion length is 12.5 mm and the pixel size is 25 μm, and thus, the spectral resolution can achieve 6.5 nm when the spectral sampling ratio is 2:1. The proposed method can provide reference for applications when appropriate materials are limited and high sensitivity is necessary.

## 1. Introduction

Spectral measurement and analysis technology has become a basic technology in the study of the composition and structure of matter, and spectrometers based on various principles have been developed one after another [1,2,3,4,5]. Among them, the fiber optic spectrometer has the advantages of low cost, high performance, small volume and weight, and it is quite convenient when used with different optic fiber probes. As a result, it is one of the most widely used types in various fields, such as scientific research, agriculture, biomedicine and industry [6,7,8].

The numerical aperture (NA) is one of the most important properties of spectral instruments, as it determines the detection sensitivity of the spectrometer [8,9]; therefore, a high NA is crucial for weak spectral signal detection applications, like fluorescent spectroscopy [9,10], Raman spectroscopy [11,12] and laser-induced breakdown spectroscopy [12,13]. In these applications, the target spectral signal is often very faint and can easily be overwhelmed by background signals. As a result, detectors such as CCDs, CMOS sensors, and InGaAs arrays must exhibit high sensitivity and typically require long integration times [14,15,16]. These factors limit the performance of fiber optic spectrometers. Increasing the NA of spectrometers represents an effective approach to enhancing sensitivity; thus, developing high NA spectrometers has become a research focus in related fields.

Currently, most commercial fiber optic spectrometers are designed based on the Czerny–Turner (CT) configuration, such as the products of Ocean Optics and Avantes. The classic CT spectrometer configuration offers advantages including low cost, simple structure, low assembling difficulty, and stable performance. However, it also presents challenges such as low numerical aperture (NA) and small field of view (FOV), due to the significant astigmatism that occurs when NA and FOV increase in CT configurations. The NA of commercial CT fiber-spectrometer (Ocean Optics, Avantes, Avenir, etc.) is usually smaller than 0.125, and the f number of CT spectrometers with long slit will be even smaller than 0.08. Some studies have proposed modified a large aperture CT configuration [17,18,19,20], but the relative aperture is still lower than 1/2.5 (corresponding to NA0.2), and at the same time, the complexity of the optical configuration will increase significantly. Curved surface grating-based spectrometer configurations like Dyson and Offner can offer high NA and FOV [21,22,23], but their expensive diffraction devices make them unsuitable for simple fiber optic spectrometers. Additionally, convex surface grating-based spectrometers are unable to achieve very high NA due to difficulties in avoiding grating occultation [23].

The transmission grating and lens-based spectrometer configuration is another choice for fiber optic spectrometer. It was first developed by Dr. Aikio [24] and is known as the prism-grating-prism (PGP) spectrometer. This kind of spectrometer configuration can achieve higher NA, as the transmission grating can work approximately in the Littrow condition; meanwhile, as high NA spectrometers usually have greater aberrations, but more lens and more optimization parameters for transmission, spectrometers make it much easier to correct all the aberrations. As a result, it is easier to acquire a high NA spectrometer with an NA of 0.33 (working f number 1.5) [25], and its volume and cost can be kept relatively low. There are also commercial fiber optic spectrometers using this transmission configuration like products of Hamamatsu [26], whose NA is 0.23. And there are also spectrometers using this configuration in other applications [27]. These transmission spectrometers all use image plane tilt to compensate the residual axial chromatic aberration, but they are all designed based on the initial configuration of an objective imaging lens, which requires apochromatism. As is known, when the spectrometer lens initial configuration is determined based on apochromatism, the subsequent optimization can hardly simplify and rectify the initial configuration, leading to redundancy in the process of determining the initial optical configuration. Therefore, if image plane tilt is only used in the process of the final optimization step, there will be redundancy of the lens in the process of determining the initial optical configuration. Consequently, achieving high performance spectrometer focusing lenses with fewer optical surfaces becomes quite challenging. This limitation severely restricts the NA or other performance indicators of transmission spectrometers.

In fact, the focusing lens of a fiber optic spectrometer has different demand compared with that of an imaging lens, and apochromatism for the fiber optic spectrometer lens design is usually unnecessary, so design simplification can be achieved. Therefore, combining with the commercially available NA 0.5 fibers, higher NA fiber optic spectrometer is also possible using the transmission grating-based spectrometer configuration.

This study presents the design principle and method of setting image plane tilt actively when finding the initial configuration of the spectrometer focusing lens, in order to increase the NA and other performance of the transmission spectrometer effectively. First, the design simplification principle of an ultra-high NA spectrometer is described, and then, a simple doublet is designed to demonstrate the advantages of the proposed method. Finally, a design example of an NA 0.5 spectrometer is presented, and its performance indicators are presented and discussed, which verifies the effectiveness of the proposed method.

## 2. Theoretical Analysis

### 2.1. The Design Principle of High NA Fiber Spectrometer

The transmission spectrometer configuration is mainly composed of three parts: the collimating lens, the transmission grating and the focusing lens. The collimating lens is used to collimate the divergent light from every point of the entrance slit, and the grating is used to diffract the polychromatic light. Finally, the focusing lens is used to image the dispersed monochromatic light onto a different position on the detector. Therefore, it can be seen that the design difficulty of a transmission spectrometer will increase as the slit gets longer and the NA gets higher.

The conventional design process for a transmission spectrometer is as follows: first the target design input parameters should be determined, such as the spectral range, spectral resolution, NA, etc. And then the focusing lens will be designed as well as an imaging lens. Once the detector is determined, the target spectral range and spectral resolution will determine the FOV and the spatial resolution of the focusing lens, and its NA should be the same as the demand. According to the design difficulty and property of various lenses’ initial configuration, an appropriate focal length and FOV can be chosen for the focusing lens, and at the same time, a grating with sufficient diffraction angles corresponding to the target spectral bands can also be determined. Next, the collimating lens can be acquired by inverting the focusing lens to simplify the system or designed separately. Finally, the collimating lens, the grating and the focusing lens are assembled together and optimized to acquire a transmission spectrometer. In addition, prisms are sometimes required to correct the spectral line smile when dealing with long silts. Furthermore, the imaging plane can also be tilt placed to further correct the residual chromatic aberration.

It can be seen from the above process that designing a high NA focusing lens is the major difficulty in designing a large aperture transmission spectrometer. Designing an NA0.5 (equivalent f number 0.87) imaging lens in a wide spectral range is a difficult task, especially as the aperture diaphragm must be the first surface of the lens, because the lens need to be integrated with the prepositive grating. However, theoretically, the focusing lens only needs to image the light of different wavelengths with different diffraction angles, so the demand of the focusing lens is not exactly the same as the imaging lens. Since the imaging plane tilt method can be used to correct the chromatic aberration of the spectrometer, we can proactively set the image plane tilt when designing the large NA focusing lens, and in this way, the chromatic aberration does not need to be corrected to approximately 0. This is a key factor to simplify the design of the high-NA spectrometer. The principle and design process will be illustrated in the next section.

### 2.2. Chromatic Aberration Correction Simplification for the Focusing Lens Design

The demand for the focusing lens used in a spectrometer can be explained from the grating equation as follows:

(1) kλ=d(sinα+sinβ)

where k is the diffraction order, λ is the wavelength, d is the grating constant, α and β are, respectively, the incident and diffraction angle of the grating. It can be easily seen that when the diffraction angle β is small, β is in direct proportion to the wavelength λ.

As the diffraction angle β of the light from the grating is just equal to the incident light angle θ of the focusing lens; then, it can be seen that the incident angle of the focusing lens is also in direct proportion to λ when θ is small:

(2) λ=t·tanθ≈tθ

where t is the simplified representation of the ratio coefficient.

**Figure 1:**
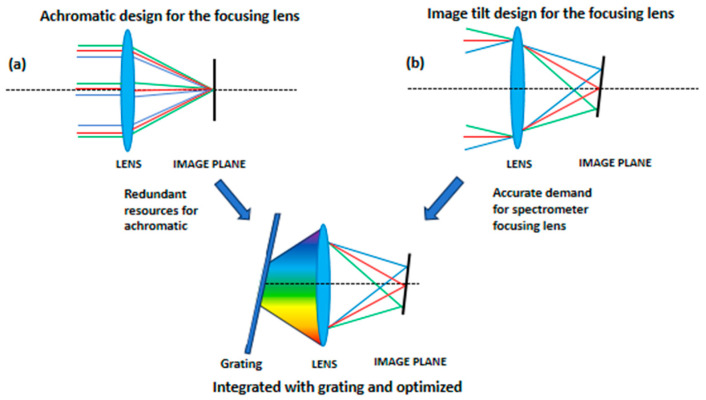
Schematic diagram of the two transmission spectrometer lens design methods. (**a**) shows the conventional method for designing an achromatic focusing lens. (**b**) shows the proposed method for designing a focusing lens using an image plane tilt, and each FOV is used to image light of corresponding wavelength.

It can be seen that the chromatic aberration correction demand for the spectrometer focusing lens is not the same as imaging lens. The focusing lens just needs to image the diffracted light of different wavelength λ with corresponding angle θ to a specific position on the image plane, and apochromatism to approximately 0 in the whole spectral range is not necessary for the focusing lens design. Therefore, the image plane tilt can be set actively during the focusing lens design process, and this will reduce the achromatism demand and simplify the design of a high NA focusing lens. The schematic diagram of the difference between the two design process is shown in Figure 1.

Next, the simplification principle of reducing the demand for chromatic aberration correction is illustrated. The normal chromatic aberration correction condition using a thin doublet of two different materials for an imaging lens can be expressed as follows:

(3) $$\varphi \left(\lambda_{1}\right)=\frac{1}{f_{\lambda_{1}}}=\varphi_{a}\left(\lambda_{1}\right)-\varphi_{b}\left(\lambda_{1}\right)=\left(n_{a}\left(\lambda_{1}\right)-1\right)c_{a}-\left(n_{b}\left(\lambda_{1}\right)-1\right)c_{b}$$

(4) $$\varphi \left(\lambda_{2}\right)=\frac{1}{f_{\lambda_{2}}}=\varphi_{a}\left(\lambda_{2}\right)-\varphi_{b}\left(\lambda_{2}\right)=\left(n_{a}\left(\lambda_{2}\right)-1\right)c_{a}-\left(n_{b}\left(\lambda_{2}\right)-1\right)c_{b}$$

(5) $$c_{a}=\frac{1}{R_{a_{1}}}-\frac{1}{R_{a_{2}}}$$

(6) $$c_{b}=-\left(\frac{1}{R_{b_{1}}}-\frac{1}{R_{b_{2}}}\right)$$

(7) $$\varphi \left(\lambda_{1}\right)-\varphi \left(\lambda_{2}\right)=\left(n_{a}\left(\lambda_{1}\right)-n_{a}\left(\lambda_{2}\right)\right)c_{a}-\left(n_{b}\left(\lambda_{1}\right)-n_{b}\left(\lambda_{2}\right)\right)c_{b}=0$$

where φ is the doublet target focal power, $n_{a}$ and $n_{b}$ are, respectively, the refractive index of the two materials, $f_{\lambda_{1}}$ and $f_{\lambda_{2}}$ are, respectively, the focal lengths corresponding to the achromatic wavelength $\lambda_{1}$ and $\lambda_{2}$. Here, we suppose that lens a is the plus lens and b is the minus lens, $\varphi_{a}\left(\lambda_{1}\right)$ and $\varphi_{b}\left(\lambda_{1}\right)$ are, respectively, the absolute value of their focal power; $R_{a_{1}}$, $R_{a_{2}}$, $R_{b_{1}}$, $R_{b_{2}}$ are the surface radius of the lens a and len b. Obviously, the focal lengths corresponding to just two wavelengths can be corrected to 0 using 2 materials, and secondary spectrum aberration remains.

When the image plane tilt is added, the ideal linear focal length $f_{l}$ of each λ should be in direct proportion to λ as follows:

(8) $$f_{l}=k_{1}\lambda +k_{2}$$

where $k_{1}$ and $k_{2}$ are the linear coefficients.

We use the difference in actual focal power φ=1/f and the ideal focal power $\varphi_{l}=1/f_{l}$ to evaluate the linearity degree of the focal length f to the wavelength λ:

(9) $$d\varphi =\varphi -\varphi_{l}=\frac{1}{f}-\frac{1}{f_{l}}=\left(n_{a}\left(\lambda \right)-1\right)c_{a}+\left(n_{b}\left(\lambda \right)-1\right)c_{b}-\frac{1}{k_{1}\lambda +k_{2}}$$

The more dφ is close to 0 in the spectral range of ($\lambda_{1}$, $\lambda_{2}$), the higher the linearity degree of actual focal length f will be. We use the Cauchy formula to describe the refractive index:

(10) $$n=A_{0}+\frac{A_{1}}{\lambda^{2}}+\frac{A_{2}}{\lambda^{4}}$$

where $A_{0}$, $A_{1}$, $A_{2}$ are, respectively, the fitting coefficients. Combining Formulas (9) and (10), the focal power linearity dφ can be expressed as follows:

(11) $$d\varphi =\frac{P_{1}}{\lambda^{4}}+\frac{P_{2}}{\lambda^{2}}+P_{3}-\frac{1}{k_{1}\lambda +k_{2}}=0$$

(12) $$P_{1}=A_{2}^{a}c_{a}+A_{2}^{b}c_{b}$$

(13) $$P_{2}=A_{1}^{a}c_{a}+A_{1}^{b}c_{b}$$

(14) $$P_{3}=A_{0}^{a}c_{a}+A_{0}^{b}c_{b}-c_{a}-c_{b}$$

It can be seen that the numerator in Equation (11) is at least a quintic equation of *λ*, there can be up to 5 solutions to Equation (11) when 2 or more lens materials are chosen rationally in the specific spectral range. It indicates that using image plane tilt when finding the initial configuration can make it much easier to design the focusing lens compared with the conventional achromatic design. When chromatic aberration for the wide spectral range can be corrected easily, the focal power of each single lens can be smaller, and the monochromatic aberration can be easier corrected. This is favorable for the large aperture system design. This will be illustrated in detail next.

### 2.3. Monochromatic Aberration Reduction for Simplified Achromatism Lens

The Seidel monochromatic aberrations for a thin lens can be expressed as follows:

(15) $$S_{1}=\frac{h^{4}\varphi^{3}}{4}\left\{\frac{n^{2}}{{\left(n-1\right)}^{2}}+\frac{n+2}{n{\left(n-1\right)}^{2}}{\left(B+\frac{2\left(n^{2}-1\right)C}{n+2}\right)}^{2}-\frac{nC^{2}}{n+2}\right\}$$

(16) $$S_{2}=-\frac{h^{2}\varphi^{2}}{2}H\left\{B\frac{n+1}{n\left(n-1\right)}+C\frac{2n+1}{n}\right\}$$

(17) $$S_{3}=H^{2}\varphi$$

(18) $$S_{4}=H^{2}\frac{\varphi }{n}$$

(19) $$S_{5}=0$$

where h is the light ray height, H is the Lagrange invariant, φ is the focal power of the thin lens, n is the refraction index, B and C are the Coddington shape factors. $S_{1}$ to $S_{5}$ represent the spherical aberration, coma, astigmatism, field curvature and the distortion, respectively.

It can be seen that the spherical aberration, coma, astigmatism and field curvature all have a positive correlation with the focal power φ. As the light ray height h and Lagrange invariant H are both larger for the small f number lens, it is important to assign the focal power of each lens to control the monochromatic aberration. 

As illustrated in Section 2.2, the achromatism requirement of spectrometer focusing lens will be weaker as the imaging plane tilt is used when finding the initial configuration. For achromatic condition of Equation (7), the ratio $\frac{c_{a}}{c_{b}}$ of the two lens curvature a and b is as follows:

(20) $$\frac{c_{a}}{c_{b}}=\frac{n_{b}\left(\lambda_{1}\right)-n_{b}\left(\lambda_{2}\right)}{n_{a}\left(\lambda_{1}\right)-n_{a}\left(\lambda_{2}\right)}$$

For the imaging plane tilt configuration, the deviation of the focal power corresponding to different wavelengths $\lambda_{1}$ and $\lambda_{2}$ is non-zero:

(21) $$\varphi \left(\lambda_{1}\right)-\varphi \left(\lambda_{2}\right)=\left(n_{a}\left(\lambda_{1}\right)-n_{a}\left(\lambda_{2}\right)\right)c_{a}^{\prime }-\left(n_{b}\left(\lambda_{1}\right)-n_{b}\left(\lambda_{2}\right)\right)c_{b}^{\prime }=\frac{1}{k_{1}\lambda_{1}+k_{2}}-\frac{1}{k_{1}\lambda_{2}+k_{2}}=k$$

where $k=\frac{1}{k_{1}\lambda_{1}+k_{2}}-\frac{1}{k_{1}\lambda_{2}+k_{2}}$ can be considered as a constant.

In this case, the ratio $\frac{c_{a}^{\prime }}{c_{b}^{\prime }}$ can be expressed as follows:

(22) $$\frac{c_{a}^{\prime }}{c_{b}^{\prime }}=\frac{n_{b}\left(\lambda_{1}\right)-n_{b}\left(\lambda_{2}\right)}{n_{a}\left(\lambda_{1}\right)-n_{a}\left(\lambda_{2}\right)}+\frac{k}{(n_{a}\left(\lambda_{1}\right)-n_{a}\left(\lambda_{2}\right))c_{b}^{\prime }}$$

As $\frac{k}{(n_{a}\left(\lambda_{1}\right)-n_{a}\left(\lambda_{2}\right))c_{b}^{\prime }}>0$, it can be infered that $\frac{c_{a}^{\prime }}{c_{b}^{\prime }}>\frac{c_{a}}{c_{b}}$. Combined with the Formulas (3) and (4), it can be seen that if the total focal power of a doublet is determined, greater curvature and focal power is necessary to achieve achromatism. When the image plane tilt is set, the plus lens and the minus lens could have lower curvature and focal power. And according to the aberration expressions (15) to (19), lower focal power means lower spherical aberration, coma, astigmatism and field curvature, which is preferable for large aperture lens design.

Therefore, setting the image plane tilt when finding the focusing lens initial configuration is necessary to acquire a large relative aperture, as the achromatism demand becomes weaker, and lower monochromatic aberration can also be achieved in this case.

### 2.4. Chromatic Doublet Design Example Using Image Plane Tilt

**Figure 2:**
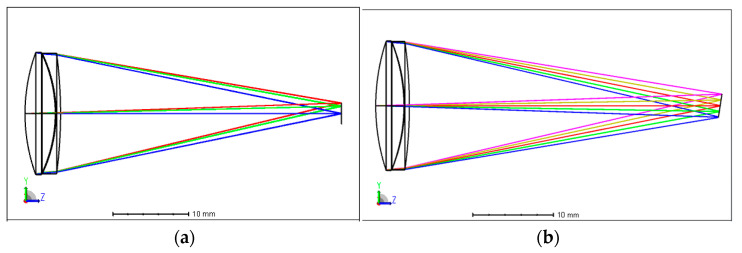
Doublet layout using achromatism and image plane tilt method. (**a**) Achromatic doublet layout. (**b**) Doublet with image plane tilt layout.

**Figure 3:**
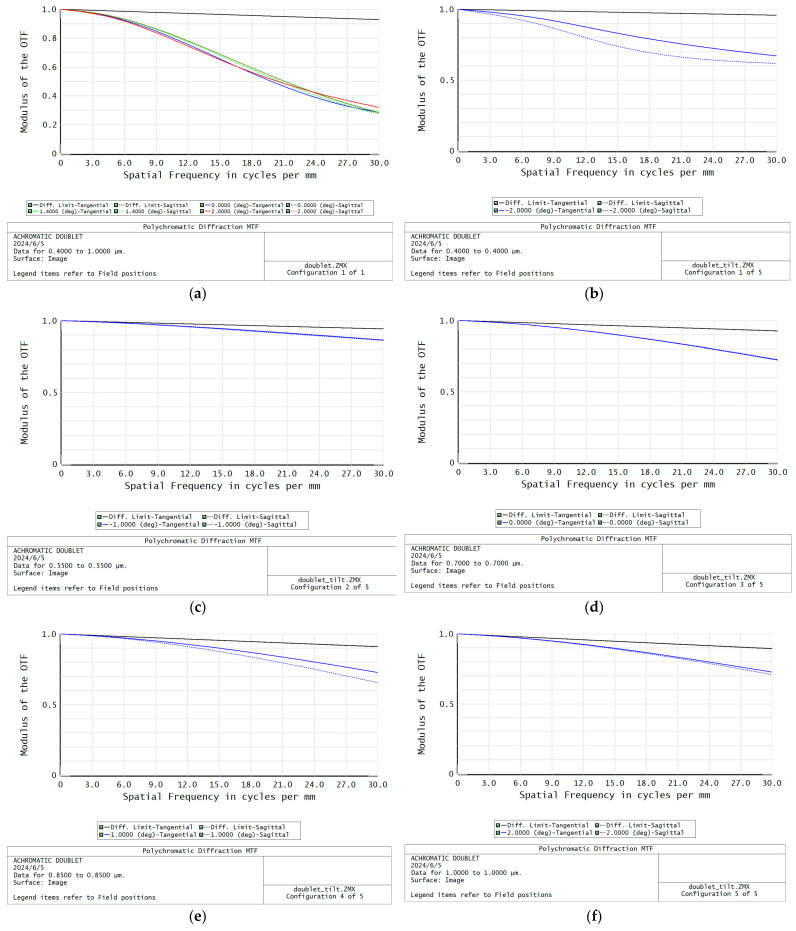
The MTF of the designed two doublets. (**a**) MTF of the achromatic doublet. (**b**–**f**) MTF of the doublet with image plane tilt: (**b**) is for −2° FOV and 0.4 μm wavelength; (**c**) is for −1° FOV and 0.55 μm wavelength; (**d**) is for 0° FOV and 0.7 μm wavelength; (**e**) is for 1° FOV and 0.85 μm wavelength; (**f**) is for 2° FOV and 1 μm wavelength.

**Figure 4:**
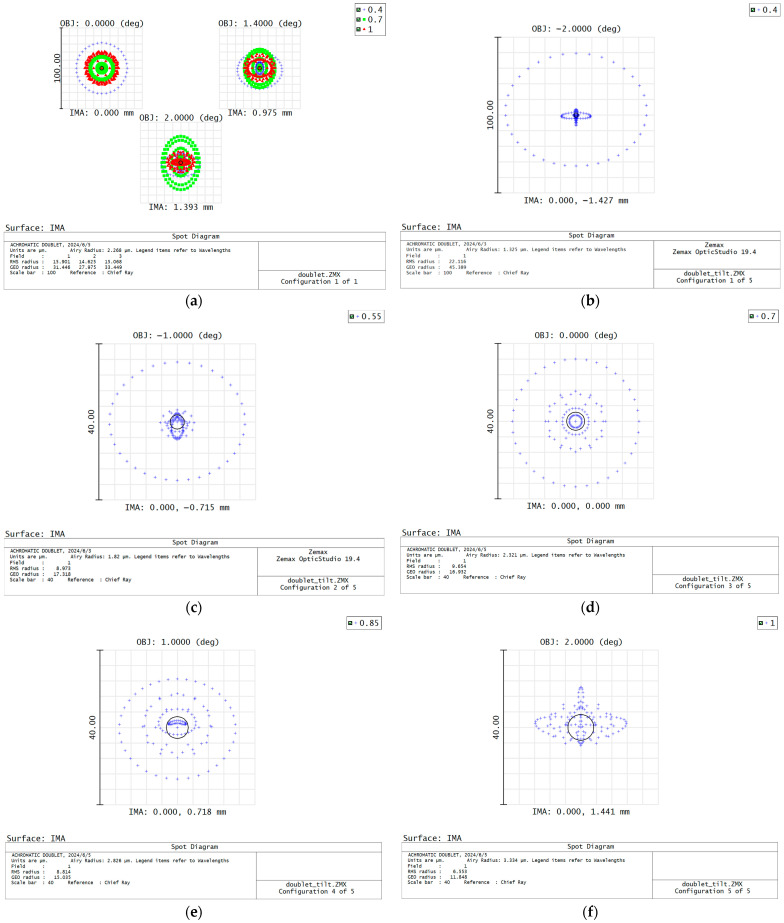
The spot diagram of the designed two doublets. (**a**) Spot diagram of the achromatic doublet. (**b**–**f**) Spot diagrams of the doublet with image plane tilt; (**b**) is the spot diagram of 0.4 μm and −2° FOV, (**c**) is the spot diagram of 0.55 μm and −1° FOV, (**d**) is the spot diagram of 0.7 μm and 0° FOV, (**e**) is the spot diagram of 0.85 μm and 1° FOV, (**f**) is the spot diagram of 1 μm and 2° FOV.

We designed a basic chromatic doublet using conventional achromatism and the image plane tilt method, respectively, to demonstrate the principle illustrated above. The doublet is composed of a BK7 plus lens and a SF2 minus lens, and the aperture is 15 mm, the focal length is 40 mm, the FOV is ±2°, and the spectral range is 400–1000 nm. The optical layouts of the designed doublets are shown in Figure 2, and the Modulation Transfer Function (MTF) and spot diagram are shown in Figure 3 and Figure 4. The parameters of the 2 doublets are shown in Table 1.

It can be seen that the MTF of conventional achromatic design can achieve about 0.3 at 30 lp/mm, with a residual secondary spectrum aberration present. The MTF of the doublet using imaging plane tilt can achieve 0.6–0.8 in all FOV, indicating that more resources can be used to correct monochromatic aberrations. The tilting angle of the doublet using imaging plane tilt is 8.13 degree; it is determined by the refractive index of the used glasses. The spot diagrams also show that using the image plane tilt can achieve better image quality for each individual wavelength and FOV. Additionally, the lens surface curvature of the doublet using the image plane tilt is lower, so it has smaller spherical aberration and comma, which is especially important for a large aperture system design. Therefore, it can be included that the method of using image plane tilt when designing the spectrometer focusing lens is helpful for high NA transmission spectrometer design.

## 3. Design Example

### 3.1. System Index and Design Process

To demonstrate the performance of the proposed design method, an NA0.5 fiber optic spectrometer with compact optical configuration is given as an example. The main design input parameters are shown in Table 2. All calculations and design processes were carried out using Zemax software version 19.4.

### 3.2. Design of the High NA Focusing Lens Using Imaging Plane Tilt

First, the optical fiber and the detector can be selected according to the NA, the target spectral range and the spectral resolution. Here, we choose Hamamatsu G11477-512WB linear InGaAs [28] (Hamamatsu Photonics, Hamamatsu, Japan) as the detector and Thorlabs NA0.5 low OH fiber (Newton, NJ, USA) as the input optical fiber.

Then, the design input of the focusing lens can be determined according to the method described in Section 2. Generally, in order to achieve the specified spectral resolution and dispersion length, two tendencies can be considered: high density grating combined with a short focal length focusing lens, or low density grating and a long focal length, small FOV focusing lens. The advantage of the former is that the total optical length of the short focal length lens will be smaller, but the disadvantage is that it is more difficult to achieve a lens with a large aperture and a large FOV, especially when the first surface is required to be the aperture diaphragm. The advantages and disadvantages of the latter solution are just the opposite.

Considering the design difficulty and system size, the focal length of the focusing lens is chosen to be 50 mm, corresponding to the ideal image plane size of 12.8 mm, and the FOV can be calculated to be approximately ±7.4°. Therefore, the design input of the focusing lens can be obtained: the FOV is ±7.4°, the working f number is 0.87, the aperture diaphragm is located at the first surface of the lens, and the target spectral range is 900–2500 nm.

Since the Petzval lens configuration has the characteristics of large relative aperture and small FOV, it is suitable for the design requirements of the focusing lens. As for designing the focusing lens, in addition to following the conventional Petzval objective lens design optimization process, according to the design simplification method described in the previous section, the focal plane is set tilted, and the Zemax multi-configuration function is used to set each lens FOV to only image its corresponding wavelength light on the image plane, and the FOV angle θ has a linear relationship with the wavelength λ. For optical materials, as the achromatic correction demand is reduced, the ZnSe and Silica with high transmission in the 900–2500 nm spectral range and good physical performance are used to simply correct the chromatic focal plane shift to a certain linear distribution. And another same material doublet is added close to the image plane as field lens to correct the residual spherical aberration and comma; meanwhile, the field curvature and astigmatism can also be corrected.

**Figure 5:**
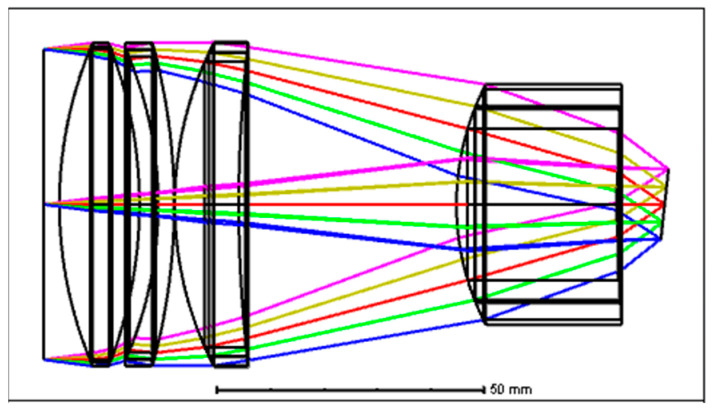
The optical layout of the designed spectrometer focusing lens.

**Figure 6:**
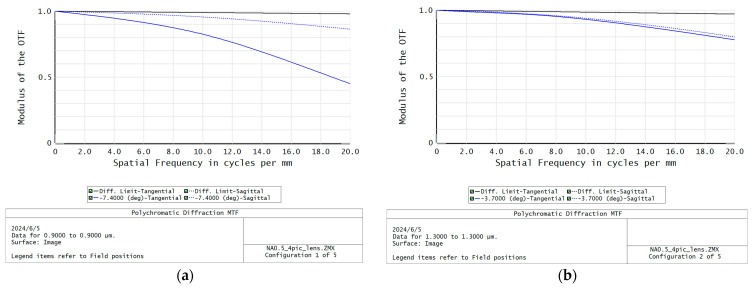

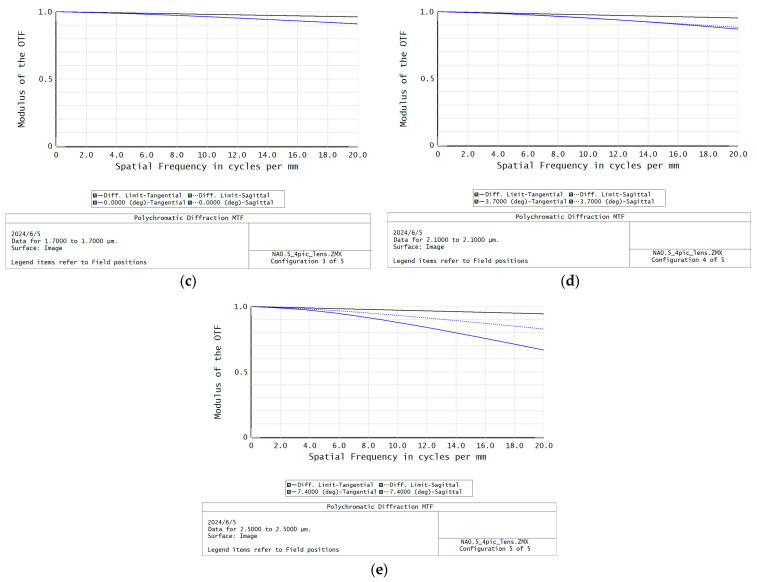
The MTF of the designed spectrometer focusing lens. (**a**–**e**) MTF of the designed focusing lens with image plane tilt: (**a**) is for −7.4° FOV and 900 nm wavelength; (**b**) is for −3.7° FOV and 1300 nm wavelength; (**c**) is for 0° FOV and 1700 nm wavelength; (**d**) is for 3.7° FOV and 2100 nm wavelength; (**e**) is for 7.4° FOV and 2500 nm wavelength.

The designed optical configuration of the focusing lens is shown in Figure 5. The MTF of each FOV corresponding to each wavelength is shown in Figure 6. It can be seen that the MTF of each FOV is higher than 0.45@20 lp/mm. When the FOV of the focusing lens is set to have a linear relationship with the light wavelengths, the design difficulty is significantly reduced, four lenses with two high efficiency optical materials are needed to meet the requirements, and pretty good imaging quality can be achieved.

### 3.3. Design of the High NA Collimating Lens Using Single Reflective Mirror

After the focusing lens design is completed, the grating density can be easily calculated based on its corresponding diffracting angles. The calculated transmission grating density is 222 lp/mm.

As the core diameter of the fiber is small, the entrance slit length is also small for fiber optic spectrometers. As is known, the paraboloid surface can collimate the light from its focus perfectly, so a single reflective paraboloid mirror can be used to collimate the incident light from the slit to simplify the system.

**Figure 7:**
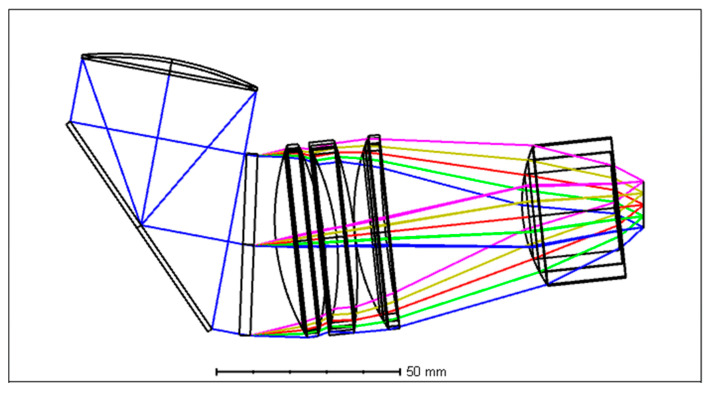
The optical layout of the designed spectrometer focusing lens.

**Figure 8:**
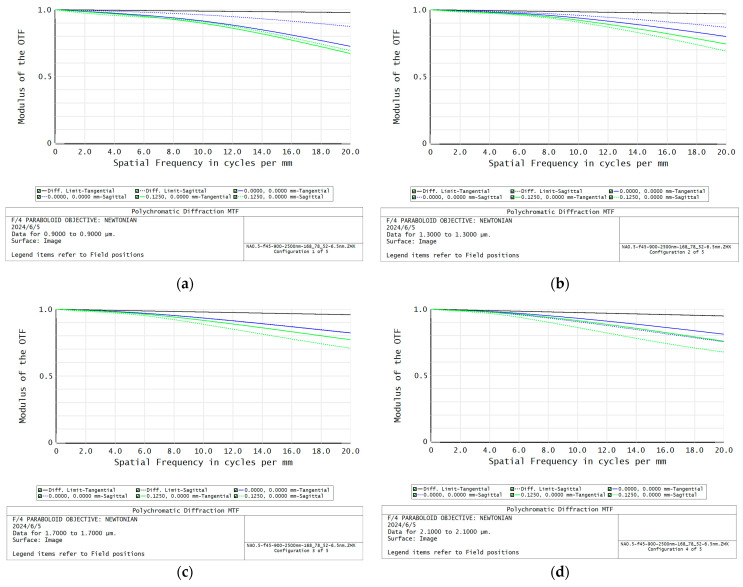

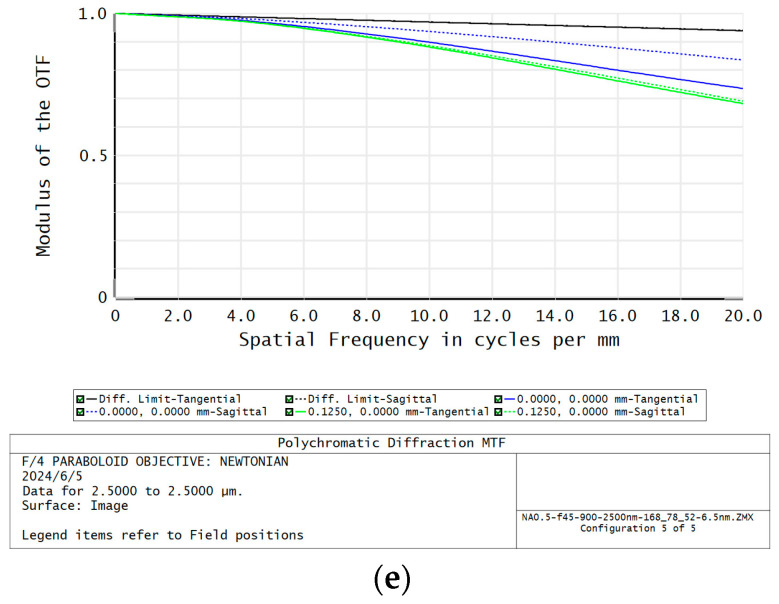
The center and edge FOV MTF of the designed NA0.5 spectrometer. (**a**) is for 900 nm wavelength; (**b**) is for 1300 nm wavelength; (**c**) is for 1700 nm wavelength; (**d**) is for 2100 nm wavelength; (**e**) is for 2500 nm wavelength.

**Figure 9:**
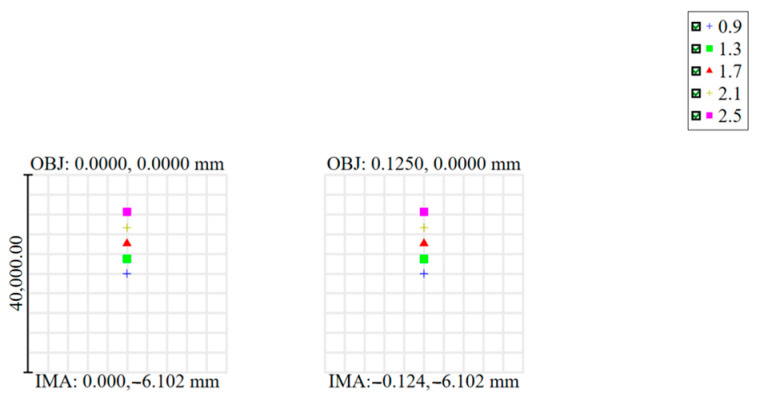
The spot diagram of the designed NA0.5 spectrometer.

To increase the sensitivity, the slit length is designed to be 0.5 mm, which is the same as the detector pixel length. A plane folding mirror with a small hole in the center has been added to simplify and adjust the process. The optical layout of the reflective paraboloid collimating lens is shown in Figure 7, and its MTF of the center and edge FOV is shown in Figure 8, and the spot diagram is shown in Figure 9. It can be seen that a single reflective paraboloid surface effectively collimates the high NA incident light from the slit.

### 3.4. Optical Layout and Design Evaluation

After the collimating lens, grating and the focusing lens are integrated and connected in series, the overall design of the high NA spectrometer is completed with slight adjustments and optimization. The optical layout of the NA0.5 spectrometer using four lenses is shown in Figure 7, and its MTF of each wavelength is shown in Figure 8, and its spot diagram is shown in Figure 9. It can be seen that the MTF of each FOV and each wavelength is higher than 0.67, indicating acceptable imaging quality. The working f number of this optical system is about 0.87, and the corresponding NA is about 0.5. The dimensions of the optical layout are 163 × 76 × 53 mm^3^; this indicates that the whole volume of the spectrometer with the InGaAs detector can also be quite compact. The spectral dispersion length is designed to be about 12.5 mm, slightly smaller than the detector size, to guarantee that all of the target spectral range can be imaged on the detector and recorded, despite the device manufacturing errors and the system assembly errors. The corresponding spectral resolution is about 6.5 nm when the pixel size is 25 μm and the sampling rate is 2:1. Therefore, it can be seen that the performance of the designed NA0.5 spectrometer can meet the input requirements. And compared to those large aperture spectrometers [22,23,24] with freeform mirrors or more than five lenses, the designed system employs simpler optical components to achieve higher NA, thus making the system relatively compact and demonstrating the effectiveness of the proposed method.

## 4. Conclusions

The spectrometer configuration based on the transmission lens has more optimization parameters in the compact structure; thus, it is easier to acquire high NA and increase compactness. However, the conventional design method for transmission spectrometers requires an initial configuration of apochromatic imaging lenses across the entire spectral range, making it challenging to design an ultra-high numerical aperture (NA) spectrometer. This necessitates the use of more lenses and a complex configuration to cover a wide spectral range and achieve high NA focusing, which is detrimental to miniaturizing the spectrometer. To date, there have been no reports of similar designs or products for ultra-high NA spectrometers.

Therefore, we propose a simplified method for designing a high NA spectrometer, and a compact NA0.5 fiber optic spectrometer design is given as an example. By deducing and analyzing the accurate requirement of the focusing lens, it can be concluded that the chromatic aberration correction demand is much weaker than that of a wide spectral range, large aperture imaging lens. When actively setting the image plane tilt during the design of the focusing lens, it is possible to greatly simplify the high NA focusing lens design. Simple doublets are designed and compared to demonstrate the performance improvement achieved by this method. Finally, an ultra-high NA spectrometer is designed, and its performance indicators show that it can achieve an NA of 0.5 with a pretty simple optical configuration, thus confirming the effectiveness of the proposed method.

As this study gives the accurate demand for the spectrometer focusing lens design, less optical materials are required, and higher performance can be achieved. This is suitable for certain spectral bands, such as near ultraviolet and shortwave infrared up to 2.5 μm, where the availability of optical materials with high performance is limited. However, more complex material combinations are still needed in the ultraviolet spectral band, as the material refractive index in the short wavelength spectral band has very strong nonlinear variation. This paper can serve as a valuable reference for researchers in related fields.

## Figures and Tables

**Table 1:** Design parameters of the two doublets.

Surface	Radius/mm (Normal)	Thickness/mm (Normal)	Radius/mm (with Image Plane Tilt)	Thickness/mm (with Image Plane Tilt)
Object	Infinity	Infinity	Infinity	Infinity
Stop	23.791	3.5	25.174	3.5
2	−18.157	0.08	−20.582	0.08
3	−18.022	0.6	−20.045	0.6
4	−59.225	37.530	−56.703	38.366
Tilting angle	0°		8.13°	
Image	Infinity	-	Infinity	-

**Table 2:** Design parameters of the high NA spectrometer.

System Indexes	Value
Spectral Range/nm	900–2500
NA	0.5
Spectral resolution/nm	6.5 nm
Sampling rate	2:1
Detector’s pixel size/μm	25 × 500

## Data Availability

No new data were created in this study. Data sharing is not applicable to this article.
